# Enteral nutrition in mechanically ventilated patients with cervical spinal cord injury

**DOI:** 10.1186/cc9798

**Published:** 2011-03-11

**Authors:** S OConnor, Y Yau, R Yandell, K Lange, J Alexander, B Freeman, M Chapman

**Affiliations:** 1 Royal Adelaide Hospital, Adelaide, South Australia, Australia; 2 University of Adelaide, Australia

## Introduction

The aim of this study was to assess the adequacy of nutrition provision to mechanically ventilated patients in the acute phase after cervical cord injury. High spinal cord injury is associated with reduced gastric emptying due to excessive sympathetic activity from the isolated thoracolumbar cord [1], which is believed to compromise nasogastric delivery of nutrition and worsen clinical outcomes. However, the success of feeding early after high spinal cord injury has not been formally evaluated.

## Methods

A retrospective cohort study. Success of enteral feeding and associated factors were reviewed for 28 days (or until ICU discharge) in all patients mechanically ventilated for at least 48 hours with cervical cord injury in a mixed, level 3 ICU, over a 2-year period. Adequacy of nutrition was defined as net calories delivered (including propofol) as a percentage of goal calories prescribed. Energy requirements were determined using the Schofield equation or a weight-based method (25 kcal/actual body weight). Data are presented as median and range.

## Results

Seventeen patients were recruited (14 male, aged 37 years (18 to 78), BMI 27 (23 to 35), APACHE II 14 (8 to 26), ASIA score A - 13, B - 4, ICU length of stay (LOS) 40 days (14 to 78), hospital LOS 82 days (34 to 219), of which two died. Six patients were discharged prior to day 28. Goal calories were 2,140/day (1,867 to 3,400). Patients commenced enteral feeding 44 hours (1 to 107) after ICU admission and received a mean 73% (SD = 19%) of nutritional goals over the 28-day study period. Energy delivery by day 4 reached 88% of goals. There was a significant relationship (*r *= 0.564; *P *= 0.029) between feed volume and hospital LOS. Feeding did not influence any other clinical outcomes including ICU LOS and mortality. Eleven (65%) patients received prokinetics for 7 days (2 to 20). No patients received TPN or post-pyloric feeding.

## Conclusions

Despite a high proportion of patients requiring prokinetics, most received adequate nasogastric nutrition during their stay in the ICU. Anecdotal evidence of weight loss and wasting after cervical spinal cord injury suggests that there are complex nutritional requirements in this group of patients and will form the basis for further studies.
